# Insulin Therapy and Cancer in Type 2 Diabetes

**DOI:** 10.5402/2012/240634

**Published:** 2012-11-14

**Authors:** Edoardo Mannucci

**Affiliations:** Agenzia Diabetologia, Ponte Nuovo, Ospedale di Careggi, Via delle Oblate, 4-50141 Firenze, Italy

## Abstract

Despite the availability of many other agents, insulin is widely used as a treatment for type 2 diabetes. In vitro, insulin stimulates the growth of cancer cells, through the interaction with insulin-like growth factor-1 (IGF-1) receptors and its own receptors. In observational surveys on type 2 diabetes, insulin therapy is associated with an increased incidence of several forms of cancer, although it is difficult to discriminate the effect of confounders from that of insulin itself. Randomized trials do not confirm the increased risk associated with insulin therapy, although they do not allow to rule out some negative effects on specific forms of cancer, at least at higher doses. Among insulin analogues, glargine has a higher affinity for the IGF-1 receptor and a greater mitogenic potency in vitro than human insulin, but it is extensively metabolized in vitro to products with low IGF-1 receptor affinity. Overall, epidemiological studies suggest a possible increase of risk with glargine, with respect to human insulin, only at high doses and for some forms of cancer (i.e., breast). Data from clinical trials do not confirm, but are still insufficient to totally exclude, such increased risk. However, beneficial effects of insulin outweigh potential cancer risks.

## 1. Introduction

Type 2 diabetes is associated with an increased incidence of malignancies and cancer-related mortality [1]. In particular, the risk of liver [1–3] and pancreas [1, 4] cancer is markedly increased in patients with type 2 diabetes, whereas other cancer types (e.g., bowel [5], stomach [6], leukemia/lymphoma [7], and breast [8]) are only moderately augmented, and other forms of malignancies (e.g., lung cancer [1]) seem to be unaffected by diabetes. As an exception, individuals with type 2 diabetes seem to be protected from prostate cancer [9]. 

The mechanisms underlying the association of type 2 diabetes with cancer are complex and not yet fully understood. The elevated incidence of hepatocellular carcinoma in patients with diabetes could be related to the common association of diabetes with nonalcoholic fatty liver disease, and with viral hepatic disease, which are both risk factors for hepatic cancer [3]. It has also been suggested that chronic exposure to hyperglycemia and hyperinsulinemia, in patients with type 2 diabetes, could contribute to the proliferation of hepatic stem cells, at higher risk for malignant transformation [10]. As for pancreatic cancer, undiagnosed neoplastic proliferation, leading to impaired insulin secretion, could facilitate the development of hyperglycemia; in this case, diabetes could be the first clinical manifestation of cancer, thus producing the reported epidemiological association [11]. 

Apart from these very specific cases, other, more general, mechanisms must be hypothesized to provide explanations for the weaker, but broader association of type 2 diabetes with several forms of cancer. Proposed mechanisms include common dietary factors, diabetes-associated adiposity (which increases the risk for several malignancies), alterations of sex hormone metabolism determined by insulin resistance and related abnormalities, and the depressive effect of chronic hyperglycemia on the immune system, which reduces its efficiency in eliminating transformed cells and controlling in situ malignancies [12, 13]. It is also possible that insulin resistance and hyperinsulinemia play a relevant role in the pathogenesis of cancer in patients with type 2 diabetes [12–14].

A relatively recent acquisition is that some of the treatments commonly used for blood glucose lowering in type 2 diabetes could affect the incidence of cancer. In particular, metformin has been reported to reduce overall cancer risk [15, 16], although results from randomized clinical trials are not fully convincing in this respect [17, 18]. On the other hand, other therapies could increase the risk of some malignancies; for example, pioglitazone treatment has been associated with a higher incidence of bladder cancer [19, 20]. Several experimental and epidemiological studies have suggested that insulin therapy could produce a substantial increase in the risk of cancer in patients with type 2 diabetes, partly explaining differences in incidence with respect to nondiabetic subjects. This paper will collect available evidence on this issue, both from preclinical and clinical studies.

## 2. Preclinical Studies: Insulin as a Growth**** Factor

Insulin is a well-known growth factors, capable of stimulating the proliferation of many cell types [21], and particularly of malignant cells [22, 23]. This effect seems to be more evident in conditions of high glucose [12, 22]. Insulin, unlike insulin-growth factor 1 (IGF-1), shows mitogenic, but not mutagenic effects, that is, it stimulates cell proliferation without inducing malignant transformation; however, it can stimulate the growth of transformed cells, facilitating their escape from immune surveillance and thus increasing the incidence rate of clinically evident tumors. On the basis of this observations, it has been suggested that a direct effect of insulin could partly explain the increased incidence of cancer in conditions of hyperinsulinemia, such as obesity and type 2 diabetes; in fact, higher levels of circulating insulin and C-peptide are associated with increased morbidity and mortality for cancer in the general population [21, 24].

The effects of insulin on cell growth can be mediated via different receptors. Insulin is a weak agonist for the IGF-1 receptor, the stimulation of which is known to promote cell growth. The affinity of insulin for this receptor is considerably lower than that of IGF-1; at the usual tissue concentrations of the two hormones, the effects of insulin on the IGF-1 receptor should be expected to be irrelevant [12, 21]. However, many cancer cells overexpress IGF-1 receptors, so that the interaction with insulin could become more relevant, particularly in conditions of hyperinsulinemia [12]. Furthermore, insulin can reduce circulating levels of IGF binding proteins 1 and 2, thus increasing concentrations of free (bioavailable) IGF-1 [25]. Apart from the interaction with IGF-1 receptors, insulin is capable of stimulating cell growth also through the binding to its own receptors, which share with those of IGF-1 part of the intracellular signaling pathways and of the biological actions. It has been observed that, during the evolutionary process, the insulin receptor was originally developed as a regulator of cell growth, There are two main types of insulin receptors, which derive from alternative splicings of the same gene transcript: type A and type B. Type A insulin receptor (IR-A) is mainly expressed during foetal and embryonic life, but it is also represented on adult cells, whereas type B (IR-B) is more typical of normal mature cells which are traditional targets for insulin metabolic actions. IR-A, which appears to mediate growth-stimulating effects more than metabolic actions of insulin, is overexpressed in many cancer cells; this is one of the reasons of the hypersensitivity of malignant cells to the growth-promoting effects of insulin [12].

The binding of insulin to both type A and type B receptors induces the phosphorylation of insulin receptor substrates IRS-1 and IRS-2 [26], which activate the phosphatidyl inositole 3 kinase (PI3K) pathway, leading to the many metabolic actions of insulin (inhibition of gluconeogenesis, expression of the GLUT-4 glucose transporter, stimulation of lipid biosynthesis, etc.). At the same time, the stimulation of PI3K activates the AKT/mTOR pathway, which promotes cell proliferation. Furthermore, stimulated insulin receptors are capable of enhancing cell growth through the activation of the MAP kinase/ERK pathway [27]. These complex mechanisms of intracellular signaling, which are at least partly shared with those of IGF-1 receptors [26, 28], promote cell proliferation as well glucose utilization and lipid synthesis. It is important to note that different types of insulin receptors activate similar intracellular pathways; in fact, IR-A, although predominantly involved in the regulation of cell growth, also mediate metabolic effects, whereas the “metabolic” IR-B have been shown to stimulate cell proliferation. In fact, in engineered cells which express only IR-B, but not IR-A and IGF-1 receptors, human insulin increases cell proliferation [29]. This means that the metabolic actions of insulin cannot be entirely separated by its growth-promoting effects.

Interestingly, in insulin resistant subjects, a greater impairment has been reported for the PI3K/AKT pathway, while the MAP kinase/ERK pathway seems to retain a greater sensitivity to insulin action in most cases; this means that insulin resistant subjects (such as those with type 2 diabetes) could be more resistant to the metabolic effects than to the growth promoting actions of insulin [12, 30]. 

## 3. Epidemiological Studies: Insulin Therapy as a Risk Factor for Cancer

Based on the results of in vitro studies, it is reasonable to suppose that insulin therapy in type 2 diabetes is associated with an increased risk of cancer. Several epidemiological (observational) studies have been performed during the past decade to verify this hypothesis. In the majority of available surveys, insulin therapy was actually associated with significantly increased risk of cancer [31–39] or a nonsignificant trend toward an increased incidence of malignancies [40–43]. A meta-analysis, which did not include some of the most recent surveys [38, 39, 43], provided an overall estimate of the increase of risk of 39% [44]. However, the results of the studies were heterogeneous, with some investigations reporting no relevant effect of insulin therapy [45–48]; in one case, a significant reduction of risk was observed in insulin-treated patients after adjusting for confounders [2].

The heterogeneity of results of available studies could reflect the biological heterogeneity of cancer. In fact, it is possible that insulin therapy is diversely associated with different cancer types. Some of the studies investigate overall cancer incidence [2, 34, 35, 37, 39, 40, 45, 46] or cancer-related mortality [32, 33], whereas others are addressed at specific cancer types, such as colorectal cancer [31, 42, 47], hepatocellular carcinoma [41, 48, 49], bladder [43], or pancreatic cancer [36, 38]. However, a wide variability of estimates can be observed even within a specific cancer type; for example, for colorectal cancer, the risk associated with insulin therapy ranges from 1.02 [47] to 2.10 [2]. The type of malignancy which seems to be more closely associated with insulin therapy in patients with type 2 diabetes is pancreatic cancer; the reported incidence in insulin-treated individuals is two- to five-fold higher than in other patients with diabetes [36, 38]. However, the majority of extra cases of pancreatic cancer in insulin-treated patients occur within a few months from the initiation of insulin therapy [36]. Considering that a pancreatic malignancy can produce an impairment of insulin secretion, deteriorating glycemic control, the prescription of insulin could be the consequence, rather than the cause, of an underlying (and still undiagnosed) pancreatic cancer. This phenomenon, known as reverse causation, could also be present for other forms of cancer, which can impair glucose control via several mechanisms, leading to an overestimation of the actual risk associated with insulin therapy. Unfortunately, very few studies [31, 39] report analyses performed with the exclusion of early cases of cancer (diagnosed in the first few months after the initiation of insulin therapy).

It should also be considered that some studies [32, 33], which happen to report a higher-than-average risk associated with insulin therapy, investigated cancer-related mortality instead of cancer incidence. It is possible that insulin therapy has a greater detrimental effect on mortality to a greater extent than incidence of cancer. In fact, in the only study in which the incidence of malignancies was reduced in insulin-treated patients, a nonsignificant trend toward an increased cancer mortality was observed, suggesting that insulin therapy could increase the lethality of cancer [2]. However, other studies have failed to detect any detrimental effect of insulin treatment on the prognosis of colorectal [50] or gastric [51] cancer.

Another major methodological limitation of epidemiological studies is represented by the effect of confounders. Patients receiving the prescription of a drug are different from those who do not receive the same prescription; observed discrepancies in the incidence of cancer could be due to the differences in the characteristics of patients, rather than to the effects of therapy. In studies performed in the general population, those receiving insulin-treatment have a remarkably higher incidence of cancer [52]; however, the large majority of those not receiving insulin are not affected by diabetes; considering that diabetes per se is a risk factor for malignancies, the difference in incidence of cancer could be due to diabetes rather than to insulin therapy. Even in the studies (cited above) in which insulin-treated patients with type 2 diabetes are compared with other, non-insulin-treated, persons with type 2 diabetes, those receiving insulin can differ in many ways from their controls: they are usually older, with a more severe form of diabetes, a higher burden of diabetic complications and comorbidities, and a poorer glycemic control—all factors which may explain, at least in part, observed differences in the incidence of cancer. Some of those confounders (e.g., age and gender) are easy to ascertain and to adjust for in statistical analyses; others can be more problematic to collect in large-scale epidemiological studies. The pattern of confounders assessed in individual studies (Table 1) is heterogeneous; in general, larger surveys based on administrative data are capable of adjusting for a smaller number of variables, and clinic-based studies with greater available detail suffer from limited sample sizes. Interestingly, among studies which assessed overall cancer incidence or cancer-related mortality, those providing higher estimates of risk with insulin therapy adjusted for a small number of confounders [32, 37, 39]. It should also be considered that, even in studies in which a greater clinical detail was available, there can be limitations in the reliability of some of the reported measures. As an example, alcohol consumption, which is known to affect the incidence of several forms of cancer, is usually collected through self-report, which is not necessarily reliable in all cases. Furthermore, there could be other, still unknown and unidentified factors which are associated with the prescription of insulin and which increase the risk of cancer. This means that the effect of confounders cannot be entirely eliminated from epidemiological studies.

Based on the considerations expressed above, it is very likely that observational studies overestimate the effect of insulin therapy on the incidence of cancer. At the same time, epidemiological studies performed in nondiabetic subjects support the hypothesis of a causative role of hyperinsulinemia in the onset of malignancies. For example, elevated plasma insulin levels are associated with a higher risk of hepatocellular carcinoma in carriers of hepatitis B virus [53], whereas higher C-peptide levels are associated with increased mortality from breast cancer [54].

In summary, epidemiological studies confirm the possible association of insulin therapy with the onset of malignancies, as suggested by experimental studies in vitro. However, this association is probably overestimated by observational surveys. Considering that epidemiological studies can be hypothesis generating, but that they cannot establish causal relationships, further evidence must be collecting through the analysis of randomized intervention trials, which do not suffer from the methodological limitations of observational studies. 

## 4. Randomized Controlled Trials: Has Insulin Any Effect on the Incidence of Cancer?

Despite the fact that insulin is the oldest available treatment, and still an important treatment option for type 2 diabetes, there are very few large-scale randomized trials comparing insulin with other treatments, which have an appropriate duration for an assessment of cancer risk. Those include the UK Prospective Diabetes Study (UKPDS), the Diabetes Mellitus Insulin-Glucose Infusion in Acute Myocardial Infarction (DIGAMI) trial, the DIGAMI-2 study, and, more recently, the Outcome Reduction with an Initial Glargine Intervention (ORIGIN) trial [55–59]. Unfortunately, the DIGAMI trial [57] did not report any data on cancer-related morbidity or mortality. Conversely, both the UKPDS [55, 56] and the DIGAMI-2 study [58] described the effects of insulin therapy on mortality from cancer, but not on the incidence of malignancies. 

In the UKPDS, the cumulative incidence of cancer-related death over 11 years similar in insulin-treated patients was similar (4.9 versus 5.0%) to that observed in other treatment groups, which included metformin, sulfonylureas, and conventional nonintensified therapy [55, 56]. In the DIGAMI-2 trial, patients with diabetes and acute myocardial infarction were randomized to intensified insulin therapy (IIT) in the acute phase and in subsequent followup (group 1), acute IIT followed by chronic conventional (mainly oral) therapy (group 2), or conventional therapy throughout the 3-year trial (group 3). The extended followup if the trial (median 4.1 year) revealed that patients randomized to chronic insulin treatment (group 1) has a significantly higher (4.4 versus 2.0%) cumulative cancer-related mortality than the other two groups [60].

The only long-term comparison of insulin with other drugs for type 2 diabetes reporting data on the incidence of malignancies is the ORIGIN trial [59]. This study enrolled 12,537 patients, the majority of whom had with type 2 diabetes, randomized to either glargine insulin or conventional (oral) therapy, with a median followup of 6.2 years. The overall incidence of cancer was identical in the two groups (Hazard ratio with 95% confidence interval: 1.00 [0.88–1.13]); similar results were reported for cancer-related mortality (HR 0.94 [0.77–1.15]). No significant difference between insulin and control group was observed in analyses for specific cancer types (breast, lung, colorectal, prostate, or melanoma).

Other large-scale, long-term trials in type 2 diabetes can provide further information on the effect of insulin therapy on cancer incidence and cancer-related mortality. The Bypass Angioplasty Revascularization Investigation 2 Diabetes (BARI 2D) study compared, in patients with acute coronary syndromes, the effect of prolonged insulin-providing (i.e., insulin and/or sulfonylureas) and insulin-sensitizing (i.e., metformin and/or thiazolidinediones) glucose-lowering therapy; the number of cancer-related deaths was similar in the two treatment groups (72 versus 70), whereas no information was provided on the incidence of malignancies [61]. There are three more trials, the action to control cardiovascular risk in diabetes (ACCORD), action in diabetes and vascular disease: preterax and diamicron modified release controlled evaluation (ADVANCE), and veteran adminstration diabetes rrial (VADT), in which intensified treatment was compared with conventional glucose-lowering therapy in patients with type 2 diabetes [62–64]. In those studies, complex algorithms including several drugs were used in both treatment arms, which differed for glycemic targets; however, the proportion of insulin-treated individuals resulted to be significantly higher in intensified treatment groups. Despite this fact, cancer-related mortality in the ACCORD trial was similar in the two arms [62], even in the longer-term (5-year) follow-up [65]. In the VADT, mortality rate for cancer was very low in both treatment groups (0.5 and 0.4% in intensified and control groups, resp., over a median followup of 5.6 years) [64]. For the ADVANCE study, data on incidence of malignancies are also available, showing no significant between-group difference [66]. 

Overall, data from randomized trial do not support the hypothesis that insulin therapy increases the risk of cancer in patients with type 2 diabetes. In fact, the only trial reporting an increased cancer-related mortality is the DIGAMI-1, while all the others, including the largest study, the ORIGIN trial [59], which collected detailed information on malignancies, did not show any increase in cancer risk. However, several reasons suggest caution in the interpretation of those data. First of all, none of the large-scale trials was specifically designed for the assessment of cancer, the principal endpoint being all-cause mortality [57], major cardiovascular events [58, 59, 61, 62, 64], or a combination of micro- and macrovascular diabetic complications [55, 56, 63]. In fact, several trials did not even report the incidence of malignancies. On the other hand, in the largest available study, the ORIGIN trial, incident cases of malignancies, although not included in the principal endpoint, were adjudicated [59]. Another inevitable limitation is represented by the fact that, in ORIGIN as well as in the UKPDS and DIGAMI-2, insulin was a rescue therapy for the control (noninsulin) groups [55, 58, 59]; as a consequence, a fraction of patients in control groups were also treated with insulin, attenuating possible between-group differences. Concomitant treatment can further confuse the interpretation of results. For example, in the ORIGIN study [59] a relevant proportion of patients in the glargine group were also treated with metformin, which has been reported to be associated with a lower incidence of cancer in insulin-treated patients [67], and which could have attenuated the effect of insulin; on the other hand, an even higher fraction of subjects was treated with metformin in the control group, possibly producing a bias against insulin. Differences in glycemic control could also be considered a confounder. Interestingly, in the only study in which glycated hemoglobin was identical across treatment groups throughout the trial (i.e., DIGAMI-2 [58]) was also the one which reported a higher cancer-related mortality in patients randomized to insulin [60]. Conversely, in the ORIGIN study and in the UKPDS, in which no difference between insulin-treated patients had a lower mean blood glucose and glycated hemoglobin than control groups [55, 59]; similar considerations can be made for BARI 2D, ACCORD, ADVANCE, and VADT [61–66]. A recent meta-analysis failed to show any effect of the improvement of glycemic control in type 2 diabetes on cancer morbidity and mortality [68]; however, the analysis was based on studies in which insulin was widely used for the intensification of diabetes therapy, possibly masking the benefits of lower glucose levels. Therefore, the possibility of a beneficial effect of a better glycemic control on the incidence and prognosis of cancer cannot be entirely ruled out.

Another issue which needs a careful consideration is that of insulin doses used in available trials. In fact, the effect of insulin on cell proliferation in vitro is dose-dependent [12] and the association of insulin therapy with cancer in epidemiological studies is related with dose and duration of treatment [69]. In the ORIGIN study, the mean insulin dose during the trial in the glargine is not reported [59]; however, considering that the median dose in patients still on insulin ranged from 0.31 (year 1) to 0.40 U/Kg∗day (year 6), and that 19% of those in the glargine group had permanently stopped insulin at year 6, the mean daily dose can be estimated in the 0.31–0.32 U/kg. This is a rather typical dose for patients on basal insulin only, but individuals on basal-bolus schemes can easily reach much higher doses. Interestingly, in the UKPDS, which reported no increase in cancer-related mortality, newly diagnosed patients were treated with long-acting human insulin only [55]; conversely, in DIGAMI-2, showing an increased mortality for cancer, patients with established diabetes received an intensified insulin treatment [58, 60]. 

Finally, different types of cancer could be diversely affected by insulin therapy, as suggested by epidemiological studies. The only trial reporting separately data on individual cancer sites, the ORIGIN study, did not highlight any relevant increase of risk for any type of malignancy [59]; however, the number of events for each kind of cancer is small, with limited statistical power.

Overall, the results of clinical trials, and particularly those of ORIGIN [59], are reassuring; however, for all the limitations discussed above, they do not allow to rule out the possibility of some negative effects of insulin. At the same time, clinical trials show that the effect of insulin therapy on the incidence of cancer, if present at all, is certainly smaller than that suggested by epidemiological studies.

## 5. Insulin Analogues versus Human Insulin: In Vitro and In Vivo Evidence

At present, insulin treatment is often based on insulin analogues, more than on human insulin. In fact, short-acting analogues, with a faster action than regular human insulin, warrant a more accurate control of postprandial peak glucose, with lower risk of hypoglycemia [70, 71]. Long-acting insulin analogues, due to the greater reproducibility of absorption, are associated with lower hypoglycemic risk than NPH human insulin [72, 73].

Insulin analogues are designed to reproduce the same biological actions of insulin, with a different kinetic profile. However, it is possible that some of these molecules differ from human insulin in some respect, thus producing undesired effects, including a higher risk for cancer. In the 1990s, the clinical development of short-acting analogue X10 was discontinued because of increased malignancies in animal models [74]. This molecule also showed an increased mitogenic potency in vitro in comparison with human insulin [75]. The difference between X10 and human insulin with respect to cell proliferation was attributed to two distinct characteristics: a higher affinity for the IGF-1 receptor [76, 77], and a lower dissociation rate from the insulin receptor [78]. The relative contribution of those two characteristics to the mitogenic effects of X10 is still unknown [75].

Of the insulin analogues presently available or under clinical development, none shows a dissociation rate from the insulin receptor different from human insulin, and only one, glargine, has an affinity for the IGF-1 receptor higher than human insulin. In vitro, glargine stimulates the growth of breast cancer cells to a greater extent than human insulin in most [79–82], but not all, cell lines [79, 80]. An increased mitogenic potency has also been observed in prostate and bowel [82], but not in thyroid [83], pancreas [84], or bladder [85] cancer cells; discordant results have been reported in osteosarcoma cell lines [80, 86]. Interestingly, glargine stimulates cell growth to a greater extent than human insulin also in engineered cells expressing only type B insulin receptors (IR-B), but not IR-A and IGF-1 receptors [29], suggesting that some of the specific mitogenic actions of this molecule could be mediated through mechanisms different from the stimulation of IGF-1 receptors.

Overall, available data show that glargine has a higher mitogenic potency than human insulin in some, but not all, cancer cell lines. However, the concentrations of insulin used in those experiments are often much higher than those reached in human plasma and tissues during insulin treatment for type 2 diabetes. Furthermore, glargine is metabolised by sequential cleavage at the carboxy terminus of the B chain, to yield products M1 and M2 [87], which both have a low affinity for the IGF-1 receptor [88]. Circulating levels of glargine during therapy are difficult to determine, because current methods do not discriminate effectively between glargine, its metabolites, and human insulin. Therefore, we do not know whether, in insulin-treated patients, glargine reaches plasma or tissue concentrations capable of affecting cancer cell growth. One pilot randomized cross-over trial showed that sera from subjects with type 1 diabetes treated with glargine stimulated the growth of human breast carcinoma cells to a greater extent than sera from the same patients during treatment with NPH human insulin [89]. This latter result suggests that circulating glargine levels during therapy are sufficient to produce effects on cell proliferation; however, the actual clinical relevance of these findings needs to be verified with in vivo studies. The use of animal models in this respect is limited by the fact that prolonged treatment with high doses of long-acting insulin formulations is associated with an elevated mortality for hypoglycemia. The best source of information, therefore, is represented by clinical studies in humans.

In 2009, an epidemiological study suggested an increased risk of overall incident malignancies in patients treated with glargine, in comparison with those receiving human insulin, after adjusting for insulin dose [35]. Several other observational surveys have been performed afterwards, with discordant results (Table 2). In fact, none of the studies reported an overall increase in the incidence of cancer in glargine-treated patients in comparison with those receiving human insulin [34, 39, 40, 45, 90–94], although one of the investigations suggested an increased risk with glargine at high doses only [90]; in one study, the incidence of malignancies with glargine was actually lower than that with human insulin [95]. When analyzing separately incident cases of breast cancer, the majority of studies found some association with glargine treatment [40, 45, 93, 95–97], although other investigations disagreed [34, 92]. Interestingly, the risk of breast cancer is more evident with higher glargine doses [93, 95] and for longer duration of treatment [96]. Furthermore, the risk for prostate cancer with glargine, as compared to human insulin, was reported to be either increased [92, 95] or unchanged [93, 96].

Overall, available observational studies suggest that glargine could be associated with an increased risk of breast and possibly prostate cancers, at least at high doses. However, the possible effect of unaccounted confounders could have interfered with results (see above). In addition, many of the studies suffer from relevant methodological limitations, such as lack of information on body mass index [35, 92–95] or insulin doses [34, 40, 45]. Furthermore, some studies [40, 45, 91, 93, 97] included patients who were already on insulin at enrolment, without information on previous exposure; of those who enrolled only insulin-naïve patients, one [34] did not exclude from analysis cases of cancer diagnosed early after the initiation of therapy. Some of the studies did not consider variations of therapy during followup [93], or used inappropriate statistical methods [35]. The mean duration of followup was lower than 4 years in all cases, except two [39, 90]. Furthermore, the duration of followup in glargine-treated patients was shorter than in comparators in the majority of studies [34, 35, 39, 92, 95, 97]. As a consequence of all these methodological limitations, the interpretation of epidemiological results is problematic. It is possible that the differences observed between glargine and human insulin are due, at least in part, to confounders or methodological biases; however, it is also possible that those biases attenuated the actual risk associated with glargine. 

Randomized clinical trials could represent a precious source of information on this issue. Unfortunately, trials comparing glargine with other long-acting insulins are usually relatively small and of short duration. The only available long-term (5 year) trial did not show any signal of concern for malignancies [98], but the sample size was too limited to confer a sufficient statistical power. A meta-analysis of trials, again showing no difference between glargine and human insulin, was composed of studies with a mean duration of less than one year [99]. Those data, such as those derived from a meta-analysis of short-term trials with the other long-acting analogue, detemir, add little to our knowledge of the relationships of those molecules with cancer risk. 

Further, and more relevant, information, can be retrieved from the results of the ORIGIN trial [59]. Although that study compared glargine with non-insulin therapies, and not with human insulin, the fact that glargine was not associated with an increased incidence of overall cancer is reassuring. However, the many reason which should induce caution in the interpretation of the ORIGIN results with respect to cancer have been already discussed above. In particular, observational studies have suggested that glargine could be associated with an increased risk only for some forms of malignancies, and only at relatively high doses. The ORIGIN study is not capable of discriminating such effects; its sample size is not sufficient to rule out possible risks for specific cancer types. For example, with respect to breast cancer (the type most frequently associated with glargine treatment in epidemiological studies), the upper limit of confidence interval of hazard ratio for glargine is 1.79 [59]—which is fully compatible with the results of observational studies. 

## 6. From Research to Everyday Practice: Clinical Implications

The possibility of an association between insulin therapy and cancer in type 2 diabetes has prompted a wide and interesting discussion in the scientific community, stimulating both basic and clinical research. The potentially alarming increase in cancer risk in insulin-treated patients suggested by many epidemiological studies, although supported by the results of in vitro investigations, has not been confirmed by randomized clinical trials. However, available trials are not sufficient to rule out the possibility of an increased risk of some specific malignancies, at least in patients receiving higher insulin doses.

This potential (and yet not demonstrated) risk must we weighed against the many well-known benefits of insulin therapy. To date, insulin remains the only therapy for type 1 diabetes, and the most effective glucose-lowering drug for type 2 diabetes [100]. An accurate glucose control is capable of preventing microvascular complications of diabetes [55, 63], and, probably, of reducing the incidence of cardiovascular disease [101–104], although results are still controversial on this point [105, 106]. Undoubtedly, benefits of glycemic control largely outweigh potential risks of malignancies associated with insulin therapy. For this reason, no patient should be prevented from receiving insulin whenever an appropriate glycemic control cannot be reached and maintained otherwise. Current guidelines, in accord with clinical practice, recommend the use of insulin in patients who are unable to reach therapeutic targets with other, non-insulin agents [100]. The prescription of insulin in type 2 diabetes is mandatory in case of ketoacidosis, or hypeglycemia associated with loss. Insulin is also recommended, even for limited periods of time, in patients with severe hyperglycemia, when a rapid reduction of glucose toxicity can be of great help in restoring beta cell function [107]. 

The main limitations of insulin therapy in type 2 diabetes are weight gain and the risk of hypoglycemia [100]; the latter has also been associated with increased cardiovascular mortality [101]. However, the use of insulin analogues has substantially reduced the hypoglycemic risk, at least at nighttime [72, 108, 109]. The improvements of insulin therapy led some authors to hypothesize an earlier use of insulin in type 2 diabetes, on the basis of the assumption that the administration of basal insulin could help in preserving endogenous insulin secretion, thus improving metabolic control and reducing the risk for diabetic complications [110]. Recently, the ORIGIN trial showed that, in comparison with a standard oral therapy based on metformin and sulfonylureas, basal insulin did not produce any beneficial effect neither on cardiovascular diseases, nor on microvascular diabetic complications, while increasing the risk of hypoglycemia and weight gain [59]; based on these results, an early use of insulin in patients with type 2 diabetes is not justified when a fair metabolic control can be reached with other drugs. In this context, the potential risks of malignancies (which were not confirmed in ORIGIN) become irrelevant for the choice of insulin therapy. 

In epidemiological studies, the risk of malignancies associated with insulin therapy is dose-dependent [69]; this result is consistent with the dose-dependent stimulation of cancer cell growth observed in vitro (see above). Based on these considerations, in insulin-treated patients it would seem rational to keep insulin doses as low as possible, provided that an adequate glycemic control is maintained. Metformin can be of help in this respect [111]; furthermore, it is possible that metformin attenuates the growth-promoting effects of insulin through its direct actions on the AMPK/mTor signaling pathway [112]. In fact, in observational studies metformin therapy in insulin-treated patients with type 2 diabetes is associated with reduced cancer incidence even after adjusting for insulin doses [67].

In summary, the risk of cancer potentially associated with insulin therapy, if present at all, should be considered irrelevant in comparison with the benefits of improved glycemic control and the other burdens of insulin treatment (most notably, hypoglycemia and weight gain). Prudentially, metformin should be associated with insulin in type 2 diabetes, unless contraindicated.

The issue of glargine insulin is more complex. The concerns arisen by studies in vitro have been only partially supported by observational studies, and they have not been confirmed by clinical trials. At the same time, trials are not sufficient to rule out the possibility of a detrimental effect of high-dose glargine on some forms of cancer. Again, an increase in risk with respect to other insulins, if present at all, is small and confined to some specific conditions. The degree of metabolic control (as measured through glycated hemoglobin) that can be obtained with NPH human insulin is not improved by the use of glargine [72, 73, 113], the only advantage being a lower risk of hypoglycemia [72, 73, 109, 113]. A similar rate of hypoglycemia, but with a marginally smaller weight gain, can be obtained with the other currently available long-acting analogue, detemir [72, 114]; however, with detemir, in comparison with glargine, a twice daily administration is required in a higher proportion of cases [114]. Furthermore, although preclinical in vitro studies are reassuring [86] and no safety issues emerged from clinical trials [115], no large-scale epidemiological surveys and no appropriately sized long-term trials are available for detemir. 

Based on these results, it is extremely difficult to provide recommendations on glargine therapy in type 2 diabetes. As a personal opinion, advantages of glargine outweigh potential cancer risks in the large majority of cases; some caution should be used with high-dose glargine, particularly in patients with previous or current breast cancer, or at high risk for that malignancy.

## Figures and Tables

**Table 1:** Epidemiological studies on the association of insulin treatment with cancer incidence or cancer-related mortality in type 2 diabetes.

Study [Ref.]	Outcome	Main result*				Confounders								
			Adiposity	Duration DM	Glucose control	Comorbidities	Medications	Ethnicity	Socioeconomic	Education	Smoking	Alcohol	Dietary factors	Physical activity
Studies included in the meta-analysis of Janghorbani et al. [44]														
Yang et al., 2004 [31]	Colorectal cancer	2.10 [1.20–3.40]	+	+	−	−	+	−	−	−	+	−	−	−
Bowker et al., 2006 [32]	Cancer mortality	1.90 [1.50–2.40]	−	−	−	+	−	−	−	−	−	−	−	−
Monami et al., 2008 [33]	Cancer mortality	2.11 [1.01–4.50]	+	+	+	+	+	−	−	−	+	+	−	−
Colhoun, 2009 [40]	All cancers	1.73 [0.98–3.05]	+	−	+	−	+	−	+	−	+	−	−	−
Currie et al., 2009 [34]	All cancers	1.42 [1.27–1.60]	+	−	+	+	+	−	−	−	+	−	−	−
Hemkens et al., 2009 [35]	All cancers	1.19 [1.09–1.29]	−	−	−	−	+	−	−	−	−	−	−	−
Jonasson et al., 2009 [45]	All cancers	1.06 [0.90–1.25]	+	+	−	+	−	−	−	+	+	−	−	−
Li et al., 2009 [36]	Pancreatic cancer	4.99 [2.59–9.61]	+	−	−	−	−	+	−	−	+	+	−	−
Monami et al., 2009 [46]	All cancers	1.01 [0.64–1.59]	+	+	+	+	+	−	−	−	+	+	−	−
Vinikoor et al., 2009 [42]	Colorectal cancer	1.74 [0.92–3.31]	+	−	−	−	+	+	+	+	+	−	+	+
Campbell et al., 2010 [47]	Colorectal cancer	1.02 [0.79–1.30]	+	−	−	−	+	+	+	+	+	+	+	+
Donadon et al., 2010 [48]	Hepatocellular carcinoma	1.24 [0.45–3.36]	+	+	+	−	+	−	−	−	−	+	−	−
Hassan et al., 2010 [41]	Hepatocellular carcinoma	1.90 [0.80–4.60]	−	−	−	−	−	−	−	+	+	+	−	−
Yang et al., 2010 [2]	All cancers	0.17 [0.09–0.32]	−	+	−	+	+	−	−	−	−	+	−	−
Baur et al., 2011 [37]	All cancers	3.87 [1.53–9.81]	+	−	+	−	−	−	−	−	+	−	−	−

Other studies														
Tseng, 2011 [43]	Bladder cancer	1.43 [0.90–2.26]	−	−	−	+	+	−	+	−	−	−	−	−
Bodmer et al., 2012 [38]	Pancreatic cancer	2.29 [1.34–3.92]	+	+	−	−	−	−	−	−	+	+	−	−
Van Staa et al., 2012 [39]	All cancers	1.79 [1.53-2.10]	+	−	−	+	+	−	−	−	+	+	−	−

**Table 2:** Epidemiological studies comparing glargine and human insulin with respect to the incidence of cancer.

Study [Ref]	Design	Comparator insulin	Main results
Hemkens et al., 2009 [35]	Cohort	Any human	Increased risk after adjusting for doses
Colhoun, 2009 [40]	Cohort	Any human	No effect on overall cancer; increased risk of breast cancer
Jonasson et al., 2009 [45]	Cohort	Any human	No effect on overall cancer; increased risk of breast cancer
Currie et al., 2009 [34]	Cohort	NPH	No effect
Mannucci et al., 2010 [90]	Case-control	NPH	No overall effect; increased overall risk for high doses (>0.3 U/kg∗day)
Ljung et al., 2011 [91]	Cohort	Any human	No effect (new short-term cohort)
Chang et al., 2011 [92]	Cohort	NPH	No effect on overall cancer; increased risk of prostate and pancreas cancer, but not of breast cancer
Morden et al., 2011 [93]	Cohort	Any human	No effect on overall cancer; increased risk of breast (but not prostate) cancer at high doses (upper quartile)
Ruiter et al., 2012 [95]	Cohort	Any human	Reduced risk of overall cancer; dose-dependent increase in the risk of breast and prostate cancer
Suissa et al., 2011 [96]	Cohort	Any human	Increased risk of breast cancer for long-term (>5 years) use
Lind et al., 2012 [97]	Cohort	Any human	Increased risk of breast cancer, dose-dependent; nonsignificant trend for prostate cancer
Van Staa et al., 2012 [39]	Cohort	NPH	No effect
Blin et al., 2012 [94]	Cohort	Human insulin	No effect
